# CYP94-mediated jasmonoyl-isoleucine hormone oxidation shapes jasmonate profiles and attenuates defence responses to *Botrytis cinerea* infection

**DOI:** 10.1093/jxb/erv190

**Published:** 2015-04-22

**Authors:** Yann Aubert, Emilie Widemann, Laurence Miesch, Franck Pinot, Thierry Heitz

**Affiliations:** ^1^Institut de Biologie Moléculaire des Plantes, Unité Propre de Recherche 2357 du Centre National de la Recherche Scientifique, Université de Strasbourg, 12 rue du Général Zimmer 67084 Strasbourg Cedex, France; ^2^Institut de Biologie Moléculaire des Plantes, Unité Propre de Recherche 2357 du Centre National de la Recherche Scientifique, Université de Strasbourg, 28 rue Goethe, 67083 Strasbourg Cedex, France; ^3^Laboratoire de Chimie Organique Synthétique, Institut de Chimie, Unité Mixte de Recherche 7177 Université de Strasbourg—Centre National de la Recherche Scientifique, 1 rue Blaise Pascal 67008 Strasbourg Cedex, France

**Keywords:** Antifungal defence, *Arabidopsis*, cytochrome P450, hormone homeostasis, jasmonate catabolism, signalling.

## Abstract

Induced resistance to the necrotrophic pathogen *Botrytis cinerea* depends on jasmonate metabolism and signalling in *Arabidopsis*. We have presented here extensive jasmonate profiling in this pathosystem and investigated the impact of the recently reported jasmonoyl-isoleucine (JA-Ile) catabolic pathway mediated by cytochrome P450 (CYP94) enzymes. Using a series of mutant and overexpressing (OE) plant lines, we showed that CYP94B3 and CYP94C1 are integral components of the fungus-induced jasmonate metabolic pathway and control the abundance of oxidized conjugated but also some unconjugated derivatives, such as sulfated 12-HSO_4_-JA. Despite causing JA-Ile overaccumulation due to impaired oxidation, *CYP94* deficiency had negligible impacts on resistance, associated with enhanced *JAZ* repressor transcript levels. In contrast, plants overexpressing (OE) CYP94B3 or CYP94C1 were enriched in 12-OH-JA-Ile or 12-COOH-JA-Ile respectively. This shift towards oxidized JA-Ile derivatives was concomitant with strongly impaired defence gene induction and reduced disease resistance. CYP94B3-OE, but unexpectedly not CYP94C1-OE, plants displayed reduced JA-Ile levels compared with the wild type, suggesting that increased susceptibility in CYP94C1-OE plants may result from changes in the hormone oxidation ratio rather than absolute changes in JA-Ile levels. Consistently, while feeding JA-Ile to seedlings triggered strong induction of JA pathway genes, induction was largely reduced or abolished after feeding with the CYP94 products 12-OH-JA-Ile and 12-COOH-JA-Ile, respectively. This trend paralleled *in vitro* pull-down assays where 12-COOH-JA-Ile was unable to promote COI1–JAZ9 co-receptor assembly. Our results highlight the dual function of CYP94B3/C1 in antimicrobial defence: by controlling hormone oxidation status for signal attenuation, these enzymes also define JA-Ile as a metabolic hub directing jasmonate profile complexity.

## Highlight

CYP94-catalysed turnover of the hormone jasmonoyl-isoleucine (JA-Ile) expands the jasmonate profile in *Botrytis*-infected *Arabidopsis* leaves and disables antifungal defence/resistance through accumulation of oxidized hormone derivatives.

## Introduction

Interaction of plants with pathogenic micro-organisms initiates cascades of molecular events in the host that are rapidly transduced into specific changes in hormone accumulation and distribution. Upon pathogen attack, typical hormones associated with induced defence such as salicylic acid (SA), ethylene (ET), and jasmonic acid (JA) undergo an increase in their abundance, due to stimulation of their biosynthetic pathways (De Vos *et al.*, 2005). In addition, other hormones such as auxin, gibberellins, abscisic acid, cytokinins, and brassinosteroids, generally linked to plant growth or development, also modulate the plant innate immune system. Pathogen attacks reconfigure plant hormonal networks where positive or negative cross-talks are optimized to induce appropriate defence responses leading to elevated resistance (Hoffmann *et al.*, 2011; Robert-Seilaniantz *et al.*, 2011; Pieterse *et al.*, 2012). For example, one way that SA suppresses JA responses is by reducing levels of the transcription factor ORA59, which targets GCC boxes in some JA-responsive promoters (Van der Does *et al.*, 2013). In addition to cross-talk, plants have evolved complex mechanisms to tightly control the timing and amplitude of individual hormonal pulses by the proper balance of biosynthesis and turnover dynamics.

The JA pathway has been studied extensively in terms of biosynthesis and signalling, mostly in defence-related but also in developmental processes (Browse, 2009; Fonseca *et al.*, 2009; Wasternack and Hause, 2013).

JA, a cyclopentanone fatty acid derivative, can be converted into a plethora of related compounds (collectively referred to as jasmonates, JAs) by oxidation, conjugation to amino acids or sugars, or other modifications (Koo and Howe, 2012; Wasternack and Hause, 2013). The co-ordinated accumulation of diverse jasmonates into a particular blend due to the action of specialized enzymes constitutes the ‘jasmonate signature’ of a given tissue. The full complement of genes/enzymes leading to this metabolic complexity, their regulation, and their biological functions still need to be elucidated.

JA signalling is a prominent example of a plant-specific paradigm of hormone signalling that proceeds through target gene derepression (Santner and Estelle, 2009). JA is a pro-hormone that acquires biological activity through Jasmonate Resistant 1 (JAR1)-catalysed conjugation towards (+)-7-*iso*-jasmonoyl-isoleucine (JA-Ile). JA-Ile directs the assembly of a co-receptor composed of the CORONATINE INSENSITIVE1 (COI1) F-box protein and JASMONATE ZIM-DOMAIN (JAZ) proteins (Chini *et al.*, 2007; Thines *et al.*, 2007; Sheard *et al.*, 2010). *Arabidopsis* possesses 12 *JAZ* genes (Chung *et al.*, 2008; Pauwels and Goossens, 2011) encoding repressors that, at a low JA-Ile concentration, block the action of transcription factors at promoters of JA-responsive genes. In conditions that stimulate jasmonate biosynthesis such as biotic stress, JAZ proteins engaged in the JA-Ile-promoted co-receptor are ubiquitinated by the SCF^COI1^ ubiquitin E3 ligase before their proteolytic elimination by the 26S proteasome, relieving the repression of target gene transcription. As *JAZ* genes are also JA-Ile responsive and stress responsive, the regulatory circuit also encodes its rapid resetting to the repressed state to prevent excessive resource allocation to defence.

In addition to the action of JAZ and other repressors on target gene promoters (Nakata *et al.*, 2013), attenuation of JA signalling also operates at the metabolic level by modification of the hormonal signal. We and others have identified members of the cytochrome P450 family (CYP94) as major actors in the catabolic turnover of the hormone during the leaf wound response (Kitaoka *et al.*, 2011; Koo *et al.*, 2011; Heitz *et al.*, 2012). CYP94B3 and CYP94C1 ω-oxidize JA-Ile at the C12 position, leading to the sequential accumulation of 12-OH-JA-Ile and 12-COOH-JA-Ile (Heitz *et al.*, 2012). As with other plant hormones (Mizutani and Ohta, 2010), there is some evidence that JA-Ile oxidation corresponds to hormonal inactivation, as plants can be rendered JA insensitive by overexpressing catabolic enzymes (Koo *et al.*, 2011; Heitz *et al.*, 2012). We recently described the existence of an additional JA-Ile turnover pathway in *Arabidopsis* wounded leaves, through the amidohydrolases IAR3 and ILL6, which cleave the JA-Ile conjugate, and also define an indirect route generating tuberonic acid (12-OH-JA). These interconnected pathways define a new metabolic grid with strong implications for jasmonate homeostasis (Widemann *et al.*, 2013).

The requirement of the JA pathway for defence against necrotrophic pathogens has been detected in various pathosystems (Penninckx *et al.*, 1998; Vijayan *et al.*, 1998). Infection by the broad-spectrum fungal pathogen *Botrytis cinerea* induces large-scale sequential changes in *Arabidopsis* genome expression that have recently been analysed with high temporal resolution (Windram *et al.*, 2012). Mutants impaired in the core JA biosynthetic pathway such as allene oxide synthase (*aos*) or 12-oxophytodienoic acid reductase (Nickstadt *et al.*, 2004; Chehab *et al.*, 2011), or in hormone perception (Thomma *et al.*, 1998), do not activate JA-dependent defences and are generally susceptible to this fungus. An *opr3 Arabidopsis* mutant was later found to be a conditional knockout (KO) that produces significant JA upon fungal infection, explaining why this line is less susceptible than *aos* mutant (Chehab *et al.*, 2011). However, recent data have independently identified 12-oxophytodienoic acid-specific regulation of callose deposition in *B. cinerea*-infected OPR3-deficient tomato plants (Scalschi *et al.*, 2015). *B. cinerea* also induces SA-dependent responses, as part of a strategy to antagonize JA-Ile-mediated resistance (El Oirdi *et al.*, 2011). It is known that distinct JA-Ile hormonal networks are operating to control inducible leaf defence against different types of attackers and are integrated by separate sets of transcription factors. MYC2 integrates JA/abscisic acid signals and defines a wound/insect-specific branch that can be probed with vegetative storage protein (*VSP*) marker induction; ERF1/ORA59 integrate JA/ET signals in a pathogen-triggered branch that is monitored by *PDF1.2* or *PR4* expression (Lorenzo *et al.*, 2004; Pre *et al.*, 2008). Both branches work antagonistically (Dombrecht *et al.*, 2007), leading to functional prioritization in subgroups of JA-Ile-regulated defences and associated resistance against microbes or insects (Lorenzo *et al.*, 2004; Nickstadt *et al.*, 2004; Verhage *et al.*, 2011).

Although many genetic factors leading to *B. cinerea* resistance are known, the way plants regulate JA-Ile homeostasis in necrotrophic pathosystems is poorly understood. For example, the diversity, abundance, and metabolic relationships of JAs accumulated in response to *B. cinerea* have not been examined in detail. The specific aim of this work was to evaluate the contribution and impact of the CYP94 catabolic pathway on microbial pathogen defence. We established the complex jasmonate profile triggered by the sequenced B05.10 strain of *B. cinerea* and showed that activation of the CYP94B3/CYP94C1-mediated JA-Ile catabolic pathway contributes to draw a unique pattern of oxidized conjugated and unconjugated jasmonates. Blocking or enhancing genetically JA-Ile oxidation respectively depleted or boosted the accumulation of the 12-OH-JA-Ile and 12-COOH-JA-Ile derivatives, but also affected the abundance of unconjugated JA derivatives. The CYP94-misexpressing lines showed a number of gene deregulations along the JA signalling and defence pathway, illustrating the role of JA-Ile oxidation in optimal antimicrobial output. Interestingly, in CYP94C1-overexpressing (C1-OE) plants, impaired defence induction and increased susceptibility to infection was observed while JA-Ile was maintained near wild-type (WT) levels, suggesting that, in this genotype, JA-Ile signalling may be non-functional due to excessive hormone oxidation.

## Materials and methods

### Plant growth and treatment

The *Arabidopsis thaliana* genotypes used were all in the Col-0 ecotype and were grown under a 12h light/12h dark photoperiod in a growth chamber. The following T-DNA insertion lines were obtained from the Nottingham *Arabidopsis* Stock Center (NASC): *cyp94b1-1* (SALK_129672), *cyp94b3-1* (CS302217), and *cyp94c1-1* (SALK_55455). The double *cyp94b3c1* mutant was obtained as described by Heitz *et al.* (2012). The triple *cyp94b1b3c1* mutant was obtained after crossing the double mutant with *cyp94b1-1* and PCR genotyping of F2 progeny. The line overexpressing *CYP94C1* (C1-OE-3B) was described previously by Heitz *et al.* (2012). The line overexpressing *CYP94B3* (B3-OE) was obtained from Dr Yves Millet (Massachussets General Hospital, Boston, USA).

For fungal infection experiments, 6-week-old plants were used. *B. cinerea* strain B05.10 was used to inoculate four to seven leaves per plant by depositing a 5 µl droplet of inoculum (2.5×10^6^ spores ml^–1^ in potato dextrose broth) on each side of the midvein. Ten inoculated plants for each genotype were placed in mini-greenhouses to ensure a high humidity until the harvest of infected leaves 2 or 3 d post-inoculation. Two leaves were collected per plant, and three pools of biological material were harvested for each time point, generating three replicates per time×genotype combination in each experiment. Leaf samples were quickly collected and flash frozen in liquid nitrogen before storing at –80 °C until analysis. All leaves (about 100 lesions per genotype) were scored for disease symptoms.

For seedling jasmonate treatment, surface-sterilized seeds were germinated in six-well microtitre plates under a 16h light/8h dark photoperiod at 22 °C in the following medium: 1× Murashige and Skoog liquid medium, supplemented with 0.5% sucrose and 0.05% MES buffer. Seedlings were grown for 7 d before gentle addition of 30 µM of synthetic JA-Ile, 12-OH-JA-Ile, or 12-COOH-JA-Ile obtained as described by Widemann *et al* (2013). At increasing time points after treatment, seedlings were quickly harvested and flash frozen in liquid nitrogen before storing at –80 °C until RNA extraction.

### *B. cinerea* relative quantification

*B. cinerea* growth in inoculated plants was determined by quantification of fungal DNA relative to plant DNA. For plant/fungal DNA extraction, 400 µl of buffer (0.2M Tris-/HCl, pH 7.5, 250mM NaCl, 25mM EDTA, 0.5 % SDS) was added to frozen leaf powder [50–70mg of fresh weight (FW)] from infected plants. The material was ground for 30 s with a Precellys 24 tissue homogenizer (Bertin Technologies, Montigny-Le-Bretonneux, France). The homogenate was cleared by centrifugation, DNA in the supernatant was precipitated with isopropanol, and the DNA was finally resuspended in 10mM Tris/HCl, pH 8. Measurement of colonization of leaves by *B. cinerea* was based on real-time PCR quantification of the fungal *CUTINASE* signal (Z69264) relative to that of *Arabidopsis ACTIN2* measured as described by Berr *et al.* (2010). Primer sequences are detailed in Supplementary Table S1 available at *JXB* online.

### Quantitative reverse transcription (RT)-PCR gene expression assays

Total RNA was extracted from plant leaves with TRIzol reagent (Molecular Research Center). One microgram of RNA was reverse transcribed using the ImProm-II reverse transcription system (Promega, Madison, WI). Real-time PCR was performed on 10ng of cDNA as described by Berr *et al.* (2010) using a LightCycler 480 II instrument (Roche Applied Science). The housekeeping genes *EXP* (At4g26410) and *TIP41* (At4g34270) were used as internal references for rosette-stage plants and *TIP41* and *GAPDH* (At1g13440) for experiments with seedlings. Gene-specific primer sequences used for quantitative RT-PCR are listed in Supplementary Table S1.

### COI1 and JAZ9 recombinant protein expression

COI1 protein was transiently expressed in *Nicotiana benthamiana* leaves. To this end, the COI1 open reading frame (At2g39940) was cloned in the *Sal*I and *Not*I sites of pENTR1a, before recombining into the pGWB20 plasmid, introducing a C-terminal 10× myc tag. The construct was transformed into *Agrobacterium tumefaciens* GV3101 strain, which was infiltrated into *N. benthamiana* leaf sectors. After 3–5 d, leaf material was harvested and stored at –80 °C until used for extraction as a tagged COI1 protein source for pull-down assay. JAZ9 cDNA was amplified and cloned in the *Bam*HI and *Not*I sites of pENTR1a plasmid, before recombining into pHMGWA plasmid, creating a His–maltose binding protein–JAZ9–His fusion protein. The construct was introduced into *Escherichia coli* BL21 strain. For protein purification, resuspended induced bacteria pellet was sonicated in lysis buffer (20mM Tris/HCl, pH 8, 300mM NaCl, 5% glycerol, 1mM EDTA, 0.1% Tween 20, 1mM PMSF, and 1mg ml^–1^ of lysozyme) on ice. The lysate was centrifuged and the supernatant was filtered (0.22 µm) before adding imidazole to 50mM. The fusion protein was purified on a HisTrap column (GE Healthcare) with an ÄTKA Purifier 10 (GE Healthcare) chromatographic system. Imidazole was used at increasing concentrations for successive column loading (50mM), washing (100mM), and elution (350mM). The imidazole was eliminated from purified protein fractions by gel filtration.

### Pull-down assay

Protein extraction and pull-down conditions were adapted from Thines *et al.* (2007). Soluble *N. benthamiana* leaf protein extract was prepared by manually grinding 1g of frozen powder in extraction buffer (50mM Tris/HCl, pH 7.5, 100mM NaCl, 25mM imidazole, 10% glycerol, 0.1% Tween 20, 20mM β-mercaptoethanol, Complete Mini Protease Inhibitor tablet EDTA-free (Roche), and 10 µM MG132). The supernatant obtained after centrifugation contained COI1–myc and was used for pull-down assays. The concentration of total protein in the extract was evaluated by the Bradford method and a BSA range.

Pull-down assays were conducted in COI1 extraction buffer in a total volume of 400 µl containing 600 µg of the total 35S::COI1–myc leaf extract and 60 µg of purified JAZ9 fusion protein. Reactions were incubated for 30min at 4 °C by gentle shaking in the absence or presence of jasmonate conjugates. One hundred microliters of Ni-NTA agarose beads was then added and the mixtures were incubated for 15min at 4 °C. After three washings with binding buffer, the beads were resuspended in elution buffer (binding buffer supplemented with 300mM imidazole). Eluates were analysed by Western blotting with anti-myc primary antibody.

### Metabolite profiling

Jasmonates and SA were identified and quantified in plant extracts by ultra-performance liquid chromatography coupled to tandem mass spectrometry (UPLC-MS/MS) as described by Heitz *et al.* (2012) and Widemann *et al.* (2013), except that the extraction solvent also contained gentisic acid as an internal standard for SA determination. SA was detected in methanol extracts with the mass transition 137>93 in negative mode. The relative quantification in samples was achieved by reporting MS peak areas relative to internal standard peak area and mass of biological material. Absolute quantifications of jasmonates were determined by comparison of the sample signal with dose–response curves established with pure compounds. Chemical synthesis of 12-OH-JA-Ile and 12-COOH-JA-Ile from commercial methyl jasmonate has been described by Widemann *et al.* (2013).

### Statistical analysis

All statistical analysis was performed using GraphPad Prism 5. Comparisons of sample means were performed by one-way analysis of variance (one-way ANOVA) and Bonferroni’s post-hoc multiple comparisons tests (*P*<0.05 for both) to determine whether sample means were significantly different.

## Results

### *B. cinerea* infection triggers a complex jasmonate profile

We established recently that plants react to leaf wounding by accumulating a blend of conjugated and/or oxidized jasmonates (Heitz *et al.*, 2012; Widemann *et al.*, 2013). In contrast to wounding, jasmonate analysis in the *Arabidopsis* response to fungi such as *B. cinerea* has generally been limited to JA quantification (Yang *et al.*, 2007; La Camera *et al.*, 2011). In particular, no information is available concerning the accumulation of the JA-Ile hormone catabolites generated by cytochrome P450 of the CYP94 family (Heitz *et al.*, 2012).

**Fig. 1.**
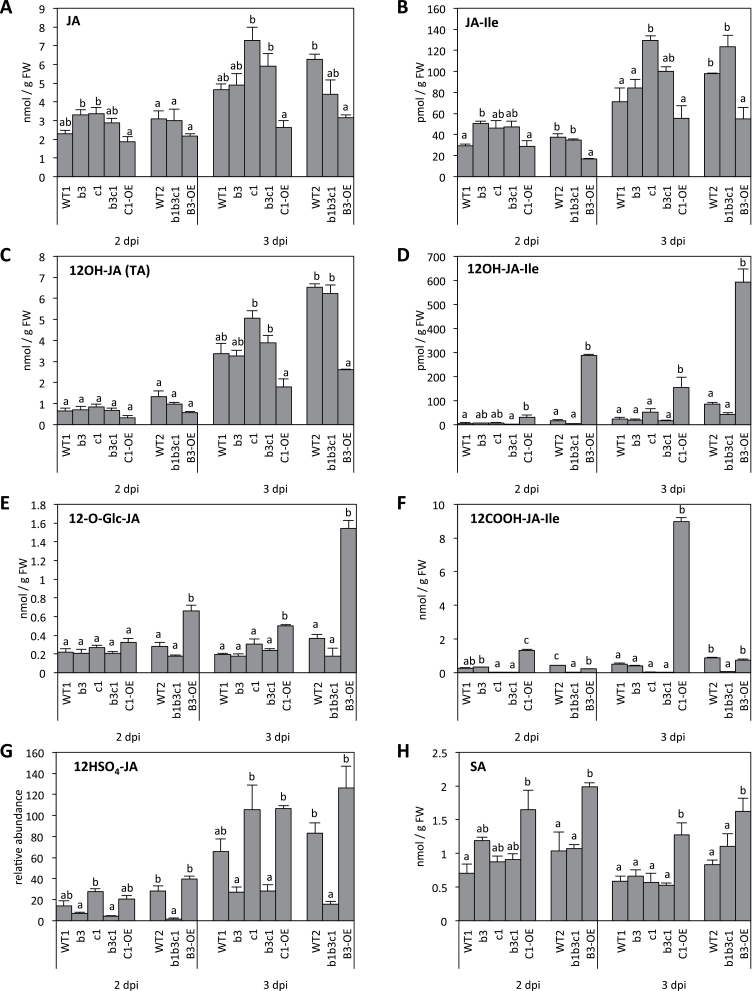
Jasmonate profiles and SA levels in *CYP94* loss- or gain-of-function mutants in response to *B. cinerea*. Six-week-old plants were drop inoculated with a suspension containing 2.5×10^6^ spores ml^–1^, leaves were harvested at 2 or 3 dpi, and jasmonates extracted and quantified by UPLC-MS/MS. (A) JA; (B) JA-Ile; (C) 12-OH-JA; (D) 12-OH-JA-Ile; (E) 12-*O*-Glc-JA; (F) 12-COOH-JA-Ile; (G) 12-HSO_4_-JA; (H) SA. Mutant genotypes are indicated as follows: *cyp94b3*, b3; *cyp94c1*, c1; *cyp94b3c1*, b3c1; *cyp94b1b3c1*, b1b3c1; CYP94B3 overexpressor, B3-OE; CYP94C1 overexpressor, C1-OE. Histograms represent the mean±SEM of six biological replicates from two independent experiments (compounds in A–D) for the block of five genotypes, or from one experiment with three biological replicates for the block of three genotypes (all compounds). Statistical analysis was applied separately at 2 or 3 dpi to blocks of five and three genotypes, each comprising a separate WT control (WT1 or WT2). Columns labelled with different letters indicate a significant difference between genotypes from a given block as determined by one-way ANOVA and Bonferroni post-tests (*P*<0.05).

WT plants were drop inoculated with fungal spores to generate two infection sites per leaf. This set-up generated enough strongly stimulated material for metabolic analysis or gene expression and allowed us to monitor plant resistance on individual lesions. Leaf extracts were prepared from infected plants and submitted to UPLC-MS/MS analysis. The spectrum of analysed jasmonates included JA, 12-OH-JA, and its glycosylated (12-*O*-Glc-JA) or sulfated (12-HSO_4_-JA) derivatives, which occur abundantly in different plant species (Gidda *et al.*, 2003; Miersch *et al.*, 2008), the hormone JA-Ile, and its two oxidation products 12-OH-JA-Ile and 12-COOH-JA-Ile (for compound structure, see Fig. 7). All JAs were close to, or below, the detection limit in leaves from uninoculated plants or even 1 d post-inoculation (dpi). Analysis was thus focused on profiling leaf extracts at 2 and 3 dpi when disease symptoms were visible. The data are presented in Fig. 1. All JAs analysed were found to accumulate at different levels upon infection. JA and its hydroxylated derivative 12-OH-JA, also known as tuberonic acid (Miersch *et al.*, 2008), were the most abundant and built up to 6–7 nmol g^–1^ of FW at 3 dpi (Fig. 1A, C). 12-OH-JA can also be further modified to 12OH-JA glucoside and 12OH-JA sulfate (Gidda *et al.*, 2003; Miersch *et al.*, 2008; Widemann *et al.*, 2013), and these compounds were readily detected in infected leaves (Fig. 1E, G). JA-Ile hormone showed maximal levels around 80–100 pmol g^–1^ of FW at 3 dpi (Fig. 1B), which is one order of magnitude lower than peak accumulation at 1h post-wounding (Heitz *et al.*, 2012; Widemann *et al.*, 2013). Finally, the JA-Ile catabolites 12-OH-JA-Ile and 12-COOH-JA-Ile were quantified at 30–90 and 600 pmol g^–1^ of FW, respectively, at 3 dpi (Fig 1D, F). Collectively, these data extend the complexity of the jasmonate profile triggered in response to *B*. *cinerea* infection, and set the framework to study the impact of JA-Ile oxidation on jasmonate homeostasis.

### Manipulating CYP94 expression impacts the jasmonate profile upon *B. cinerea* infection

**Fig. 2.**
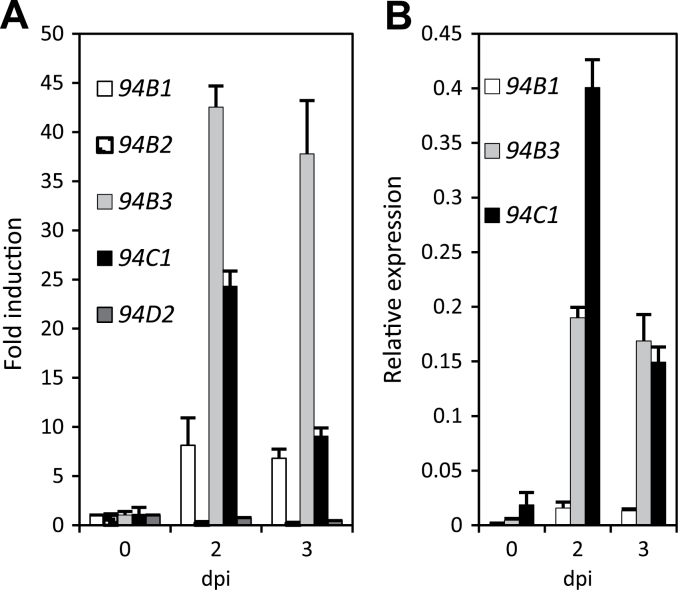
*CYP94* gene expression in WT plants in response to *B. cinerea*. WT (Col-0) leaves were harvested prior to infection (0 dpi) and 2 or 3 dpi. Expression of *CYP94* genes was determined by real-time PCR and normalized using *EXP* (At4g26410.1) and *TIP41* (At3g18780.2) as reference genes. (A) Expression profiles of *CYP94* genes in response to *B. cinerea* infection. Expression is represented as fold induction relative to expression level at T0, which was set to 1 for each gene. (B) Relative expression of *CYP94B1*, *CYP94B3*, and *CY 94C1* genes, represented as the gene-specific signal corrected by the reference gene signal. Histograms represent the mean±SEM of three technical replicates. The experiment was repeated independently and produced similar results.

JA-Ile oxidized derivatives are formed upon mechanical wounding due to the transcriptional upregulation of two *CYP94* genes, *CYP94B3* and *CYP94C1* (Kitaoka *et al.*, 2011; Koo *et al.*, 2011; Heitz *et al.*, 2012). The accumulation of these oxidation products after fungal infection prompted us to investigate the expression of the six *CYP94* genes in this pathosystem. *CYP94D1* presents features of a pseudogene (Y. Aubert, unpublished data) and its transcripts were undetectable in leaves. *CYP94B2* and *CYP94D2* showed weak or moderate expression, respectively, in control leaves, which declined in diseased leaves (Fig. 2A). Three genes, *CYP94B1*, *CYP94B3*, and *CYP94C1* were clearly upregulated, with *CYP94B3* showing the highest fold change compared with unstimulated leaves (Fig. 2A, T0 set to 1 for each gene), due to its very low basal expression level. When data were calculated as expression levels (without fixing the T0 value, with the target signal corrected by the signal of two reference genes), *CYP94C1* appeared as the most expressed member, peaking at 2 dpi, followed by *CYP94B3*, and then by the much lower expression of *CYP94B1* (Fig. 2B). The expression dynamics of the *CYP94B3* and *CYP94C1* genes were therefore most consistent with a role in the formation of oxidized JA-Ile conjugates in infected tissues. We also determined that the second pathway for JA-Ile turnover in wounded leaves (Widemann *et al.*, 2013), involving the amidohydrolases IAR3 and ILL6, was also upregulated in infected leaves, with *IAR3* showing the strongest response (Supplementary Fig. S1 available at *JXB* online).

We next used a set of single, double, and triple mutants in *CYP94B1*, *CYP94B3*, and *CYP94C1* genes (Heitz *et al.*, 2012) to dissect their contribution to JA-Ile oxidation and more generally to JA homeostasis upon *B. cinerea* infection. The seven plant genotypes were the previously described single *cyp94b3* and *cyp94c1*, and double *cyp94b3c1* mutants, and a line ectopically overexpressing *CYP94C1* (C1-OE) under the cauliflower mosaic virus 35S promoter (Heitz *et al.*, 2012), along with the newly generated *CYP94B3* overexpressor (B3-OE) or *cyp94b1b3c1* triple mutant. The expression characteristics of these latter lines are described in Supplementary Fig. S2 available at *JXB* online. Infected leaves were collected at 2 and 3 dpi, methanol extracts were submitted to UPLC-MS jasmonate profiling, and the abundance of JAs was compared among genotypes as presented in Fig. 1. Because of the large number of plant populations handled in the study, we treated them in parallel as two separate sets of plants each including a WT control population (WT1 and WT2). Mutants impaired in JA-Ile oxidase activity were expected primarily to display changes in JA-Ile and related conjugates; however, pathway interruption redirects flux to alternative routes and consequently may affect the abundance of more distant compounds, as reported previously (Heitz *et al.*, 2012; Widemann *et al.*, 2013). The levels of JA, the most upstream pathway compound analysed (Fig. 1A), were enhanced in *cyp94* mutants and were reduced by 30–40% in both OE lines at 3 dpi, consistent with altered JA consumption in these lines. JA-Ile, the endogenous CYP94 substrate, was also differentially affected by CYP94 mis-expression: its levels were increased as expected in most KO lines at 2 and 3 dpi, and were reduced by about 50% in B3-OE, in accordance with enhanced hormone turnover. In contrast, JA-Ile content appeared similar to that of the WT in the C1-OE line, as established in two independent experiments (Fig. 1B). The accumulation of JA-Ile oxidation products was found to be largely affected by CYP94 depletion or overexpression (Fig. 1D, F). 12-OH-JA-Ile, the major *in vitro* product of CYP94B3 activity (Heitz *et al.*, 2012) but the least abundant compound analysed here, was reduced in the double and triple mutant lines (Fig. 1D, 2 dpi) compared with the WT, and was enhanced 6–15-fold by *CYP94B3* overexpression, or 5-fold by *CYP94C1* overexpression (Fig. 1D). In addition, levels of 12-COOH-JA-Ile, the major *in vitro* product of CYP94C1 activity (Heitz *et al.*, 2012), were nearly undetectable in all *cyp94c1*-deficient lines (Fig. 1F), and were boosted 15-fold over the WT in the C1-OE line at 3 dpi. These data demonstrated the primary role of CYP94B3 and CYP94C1 in the accumulation of oxidized derivatives of the JA-Ile hormone in response to *B. cinerea* infection. We showed previously that, upon leaf wounding, 12-*O*-Glc-JA and 12-HSO_4_-JA derive from 12-OH-JA (tuberonic acid) generated through 12-OH-JA-Ile cleavage by the amidohydrolases IAR3 and ILL6 (Widemann *et al.*, 2013). In response to fungal infection, 12-OH-JA levels were only weakly affected in single or multiple *cyp94* mutants, but were decreased in OE lines, most significantly in B3-OE at 3 dpi (Fig. 1C). The two 12-OH-JA derivatives were accumulated after fungal infection, with much higher 12-*O*-Glc-JA levels in both OE lines (Fig. 1E, G), but were differentially affected by *CYP94* mutations. 12-*O*-Glc-JA was only moderately reduced in the triple *cyp94b1b3c1* mutant, whereas 12-HSO_4_-JA accumulation was strongly impaired in all CYP94B3-deficient lines, indicating distinct precursor trafficking for these two 12-OH-JA derivatives.

### CYP94-mediated JA-Ile catabolism impacts *B. cinerea* resistance responses

**Fig. 3.**
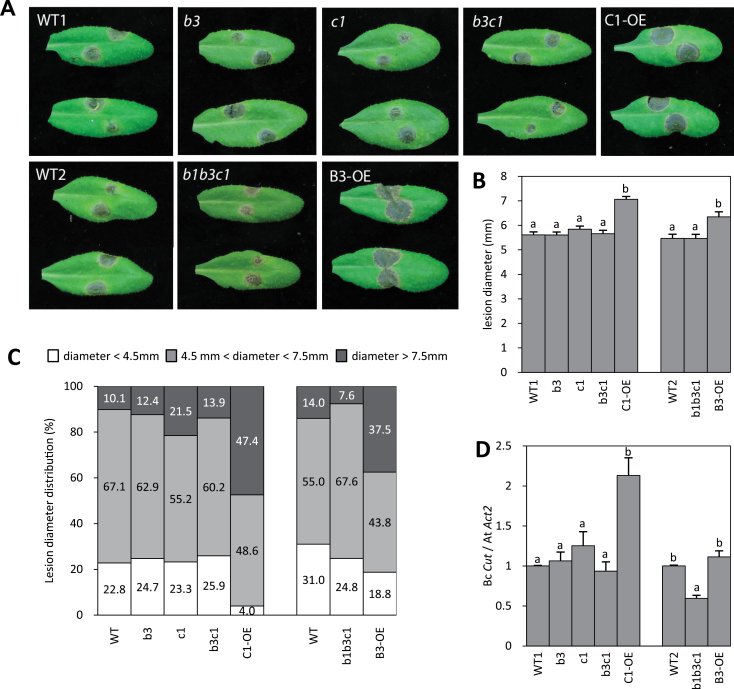
Resistance levels to *B. cinerea* of *CYP94* loss- or gain-of-function mutants. Disease symptoms from plants analysed in Fig. 1 were scored. (A) Visual aspect of lesions sites at 3 dpi. (B) Mean lesion diameter at 3 dpi. Histograms represent the mean lesion diameters±SEM of at least 100 lesion sites from 10 plants for each genotype. (C) Scoring of symptoms by determination of the distribution of lesions in three diameter classes: small: <45mm; medium: 45–75mm; and large: >75mm. (D) Quantification of fungal growth by real-time PCR on *Botrytis* genomic DNA with *B. cinerea* cutinase-specific primers at 3 dpi. Histograms represent the mean±SEM from two independent experiments for the block of five genotypes, or from one experiment for the block of three genotypes. In each individual experiment, fungal DNA quantification was performed on three biological replicates analysed in triplicate. Statistical analysis was applied to blocks of five and three genotypes each with a separate WT control (WT1 or WT2). Columns labelled with different letters in (B) and (D) indicate a significant difference as determined by one-way ANOVA and Bonferroni post-tests (*P*<0.05). (This figure is available in colour at *JXB* online.)

We then examined whether these metabolic alterations, particularly those related to JA-Ile levels, had an impact on *B. cinerea* resistance. Representative disease symptoms recorded at 3 dpi on the different genotypes are shown in Fig. 3A. Lesion diameters were scored as shown in Fig. 3B, and their size distribution was established for all genotypes (Fig. 3C). Average lesion sizes were not significantly different from the WT in mutant plants, but B3-OE and C1-OE plant lines displayed much larger lesions, characteristic of a reduced resistance phenotype (Fig. 3A, B). Both OE lines displayed more large (>7.5mm) lesions (Fig. 3C). To determine whether changes in lesion size were due to altered fungal growth, we monitored fungal DNA content in leaves by real-time PCR by amplifying a *B. cinerea* genomic cutinase fragment. Consistent with symptom severity, fungal DNA was 2.5-fold more abundant in the C1-OE line at 3 dpi (Fig. 3D). In contrast, the fungal DNA content was found to be increased, but not significantly, in the B3-OE line, but reduced in the triple mutant. Collectively, these data indicated that *CYP94C1* and *CYP94B3* expression positively correlated with stronger disease symptoms and/or growth of *B. cinerea*.

### CYP94-mediated JA-Ile catabolism impacts defence responses to *B. cinerea*

**Fig. 4.**
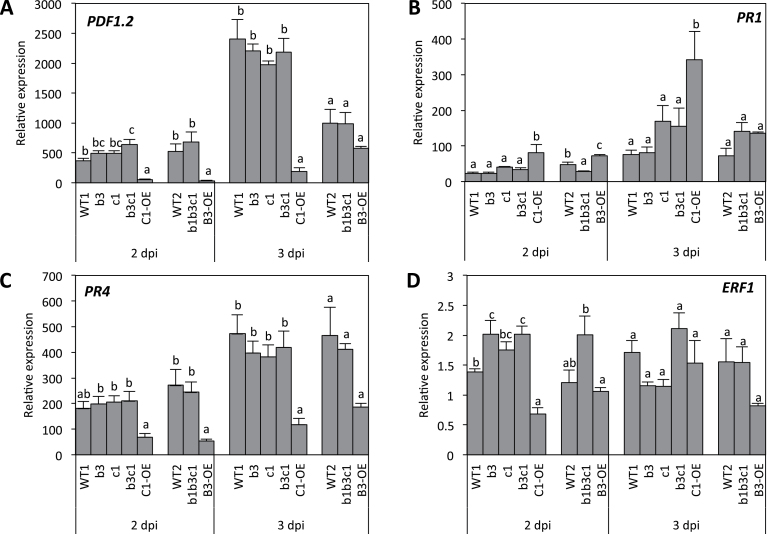
Expression profiles of jasmonate-dependent defence genes in response to *B. cinerea* in *CYP94* loss- or gain-of-function mutants. Six-week-old plant were drop inoculated with a suspension containing 2.5×10^6^ spores ml^–1^, leaves were either harvested prior inoculation or at 2 or 3 dpi. Expression of *PDF1.2* (A), *PR1* (B), *PR4* (C), and *ERF1* (D) was determined by real-time PCR using gene-specific primers and normalized using *EXP* and *TIP41* as reference genes. Histograms show the means±SEM either of two independent experiments (block of five genotypes) or from one experiment (block of three genotypes). In all individual experiments, transcript quantification was performed on three biological replicates analysed in duplicate. Expression is represented as relative expression of the target gene relative to its expression level at 0 dpi corrected by the two reference genes [and set to 1 in (B)]. Statistical analysis was applied separately at 2 or 3 dpi to blocks of five and three genotypes each with a separate WT control (WT1 or WT2). Columns labelled with different letters indicate a significant difference between genotypes as determined by one-way ANOVA and Bonferroni post-tests (*P*<0.05).

Because of changes in JA profiles and resistance levels to *B. cinerea* infection, we investigated *CYP94*-modified plants for the transcriptional behaviour of marker genes in the defence cascade. *PDF1.2* and *PR4* encode antifungal proteins and probe the activation of the antimicrobial branch of the JA pathway (Pieterse *et al.*, 2012; Wasternack and Hause, 2013). Expression of both genes was readily increased in response to *B. cinerea*, with a slight enhancement in *cyp94* mutants at 2 dpi compared with the WT (Fig. 4A, C). In contrast, both *PDF1.2* and *PR4* expression was strongly impaired in OE lines at both collection times. In particular, at 2 dpi, *PDF1.2* expression was barely detectable in these lines. *B. cinerea* is countered by the induction of ET/JA-dependent defence responses (Thomma *et al.*, 1998), but this fungus also induces to a lower extent the SA-dependent pathway, known to be more potent in defending *Arabidopsis* against biotrophic pathogens (Pieterse *et al.*, 2012). The SA-marker *PR1* was more induced in OE lines, mostly in C1-OE (Fig. 4B), a response that was correlated with higher free SA levels (Fig. 1H), suggesting that excessive JA-Ile oxidation relieves the SA pathway that is restrained in the WT.

*ERF1* encodes a transcription factor positively regulating *PDF1.2* and *PR4* expression (Lorenzo *et al.*, 2004). We investigated whether defence gene misregulation was associated with perturbations in *ERF1* expression. *ERF1* early expression (2 dpi) was enhanced in all *CYP94*-deficient lines (Fig. 4D), and dropped in single mutants at 3 dpi. Conversely, *ERF1* transcripts were reduced in both OE lines: C1-OE at 2 dpi and B3-OE at 3 dpi. These results showed that CYP94B3/C1-encoded JA-Ile oxidation transiently distorts *ERF1* expression, which only partially correlates with changes in *PDF1.2* and *PR4* expression.

**Fig. 5.**
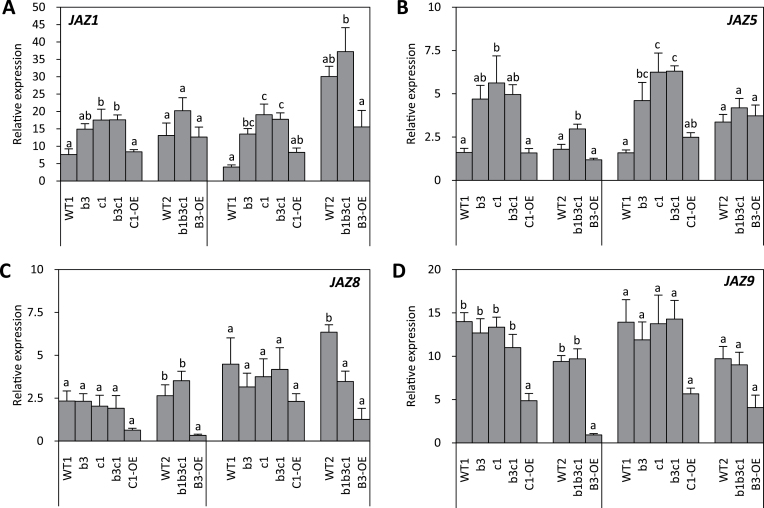
Expression profiles of JAZ repressors in response to *B. cinerea* in *CYP94* loss- or gain-of-function mutants. cDNA was prepared as described in Fig. 4 and expression of *JAZ1* (A), *JAZ5* (B), *JAZ8* (C), and *JAZ9* (D) was determined by real-time PCR using gene-specific primers and normalized with *EXP* and *TIP41* as the reference genes. Histograms represent the means±SEM of two independent experiments (block of five genotypes) or from one experiment (block of three genotypes). In all individual experiments, transcript quantification was performed on three biological replicates analysed in duplicate. Expression is represented as relative expression of the target gene relative to its expression level at 0 dpi corrected by the two reference genes [and set to 1 in (A)]. Statistical analysis was applied as in Fig. 4.

In an attempt to understand why increased JA-Ile levels and *ERF1* expression did not result in enhanced defence gene expression in *cyp94* KO lines, we evaluated the expression of *JAZ* genes as signalling components repressing the JA response (Chini *et al.*, 2007; Thines *et al.*, 2007; Yan *et al.*, 2007). JA-Ile signalling and gene derepression relies primarily on pre-existing JAZ protein degradation, but strong transcriptional *JAZ* gene upregulation is believed to restore target gene repression during the signal attenuation phase. Among the 12 *Arabidopsis JAZ* genes, most have been shown to be responsive to wounding (Chung *et al.*, 2008) or to *Pseudomonas syringae* bacterial infection (Demianski *et al.*, 2012). We showed here that *JAZ1, -5, -6, 7, -8, -9 and -10* were also induced to various extents in WT plants by *B. cinerea* infection (Supplementary Fig. S3 available at *JXB* online). *JAZ8* exhibited the strongest induction factor relative to T0, due to an extremely low basal level (Supplementary Fig. S3). When the *JAZ* signal was normalized only with the reference gene signal, *JAZ9* was the most expressed gene in infected leaves (Supplementary Fig. S3B). We analysed the behaviour of four of these genes in *CYP94*-modified lines. *JAZ1* (Fig. 5A) and *JAZ5* (Fig. 5B) displayed WT expression in both OE lines but showed a strong overinduction trend in mutant lines. In marked contrast, *JAZ8* and *JAZ9* expression was largely unaffected by *cyp94* mutations, and was impaired in both OE lines. These data illustrated that individual *JAZ* repressor genes are differentially affected by the plant capacity to oxidize the JA-Ile hormone.

### Oxidized JA-Ile derivatives are unable to induce JA-responsive gene expression

**Fig. 6.**
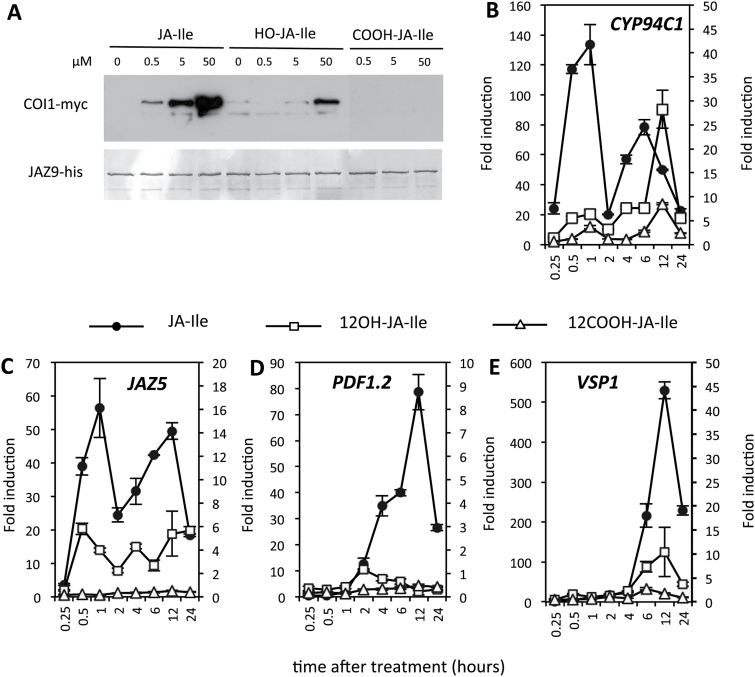
Sequential JA-Ile ω-oxidization abolishes the capacity to promote co-receptor assembly and induction of jasmonate-responsive genes. (A) *In vitro* pull-down assay performed with purified His-tagged JAZ9 and *N. benthamiana* leaf extract expressing AtCOI1–myc, in the presence of increasing concentrations of JA-Ile, 12-OH-JA-Ile, or 12-COOH-JA-Ile as indicated. JAZ9 Coomassie blue staining on the membrane is shown on the lower panel to indicate equal recovery and loading. (B–E) Seven-day-old liquid-grown seedlings were incubated in the presence of 30 µM JA-Ile, 12-OH-JA-Ile, or 12-COOH-JA-Ile for the indicated time periods. Seedlings were flash frozen in liquid nitrogen and submitted to RNA extraction. One microgram of total RNA was reverse transcribed and expression of *CYP94C1* (B), *JAZ5* (C), *PDF1.2* (D), and *VSP1* (E) was determined by real-time PCR using gene-specific primers and normalized with *GAPDH* and *TIP41* as the reference genes. Histograms represent the means±SEM of three technical replicates. Expression is represented as fold induction relative to expression level at T0, which was set to 1 for each gene. Fold induction by JA-Ile (black circles) is shown on the left scale of each graph and fold induction of oxidized conjugates (12-OH-JA-Ile, open squares; 12-COOH-JA-Ile, open triangles) is shown on the right scale of each graph. Mock treatments induced no response and are not shown for clarity. The experiment was repeated and generated similar results.

The monitoring of gene expression at different levels in the JA signalling cascade revealed that, despite moderate alterations in JA-Ile levels, *CYP94*-modified plants exhibited strong perturbations in the downstream output of the JA defence pathway. In particular, B3-OE and 94C1-OE plants, which both exhibited impaired *B. cinerea* resistance, had particular jasmonate profiles characterized by the overaccumulation of 12-OH-JA-Ile and 12-COOH-JA-Ile derivatives, respectively, that could influence defence signalling. To compare their capacity to promote *in vitro* JAZ–COI1 co-receptor assembly, the initial event in JA-Ile signalling, we assayed the three JA-Ile conjugates in a pull-down assay using tagged versions of COI1 and JAZ9 (the most expressed JAZ protein in response to *B. cinerea*). JA-Ile promoted a dose-dependent assembly of the co-receptor, while 12-OH-JA-Ile generated only a weak response at 50 µM (Fig. 6A), consistent with a previous report (Koo *et al.*, 2011). Under these conditions, 12-COOH-JA-Ile produced no detectable signal, indicating that the most oxidized JA-Ile conjugate was devoid of activity in this assay.

To further assess individual signalling properties of JA-Ile oxidized derivatives, we tested their gene-inducing activity by external application to 7-d-old liquid-grown seedlings. This system allows a direct and continuous contact with the inducer without inflicting undesired stress, and results in high-amplitude responses. JA-Ile, 12-OH-JA-Ile, or 12-COOH-JA-Ile (30 µM) was applied and plantlets were analysed in kinetic studies for the transcriptional responses of pathway marker genes. JA-Ile readily induced the early and biphasic (peaks at 0.5–1h and 6–12h) expression of *CYP94C1* (Fig. 6B) and *JAZ5* (Fig. 6C), while *PDF1.2* and *VSP1* exhibited a delayed response as expected for late marker genes (Fig. 6D, E). Comparatively, both oxidized compounds led to weak or no responses (scales on right of graphs in Fig. 6B–E). More precisely, 12-OH-JA-Ile provoked detectable responses, ranging from 1/50 of the JA-Ile response for the MYC2-target *VSP1* to 1/5 for *CYP94C1* at 12h, while no *PDF1.2* induction was detectable. In contrast, 12-COOH-JA-Ile was unable to induce any of the genes analysed. These data indicated that sequential JA-Ile oxidation by CYP94 enzymes resulted in an incremental decrease in hormonal activity.

## Discussion

### *Botrytis* infection induces a complex array of jasmonate derivatives

**Fig. 7.**
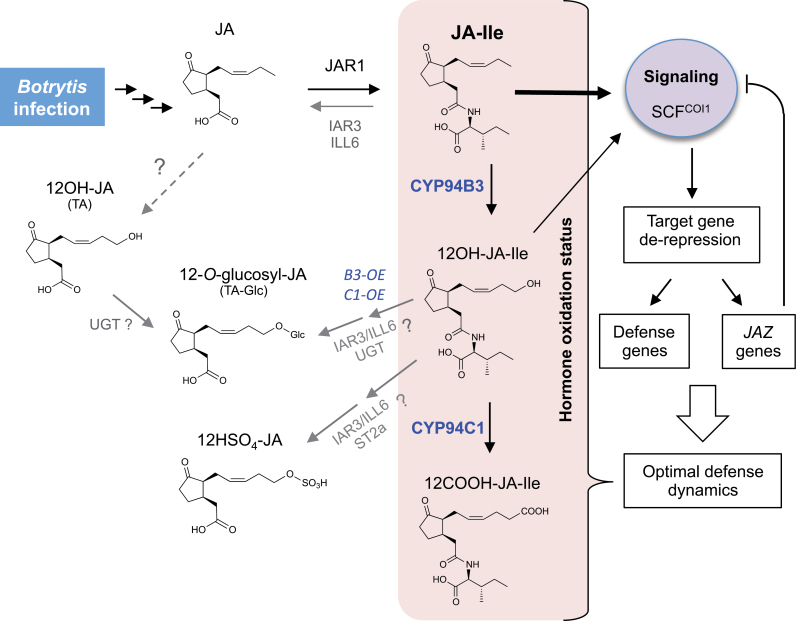
Working model of metabolic conversions in the jasmonate pathway upon *Botrytis* infection. All represented jasmonates accumulate in infected leaves, but with distinct pool sizes and possible differences in major routes compared with the wound response (Widemann *et al.*, 2013). The hormone JA-Ile is oxidized/inactivated to 12-OH-JA-Ile and 12-COOH-JA-Ile by the CYP94B3 and CYP94C1 enzymes. Additional conversion routes were inferred after profiling CYP94 KO or OE lines. Tuberonic acid (12-OH-JA; TA) and 12-*O*-Glc-JA seem to be formed in infected WT plants by unknown enzymes (dashed line) with a minor contribution of CYP94 activity. In contrast, accumulation of 12-HSO_4_-JA is strongly CYP94B3 dependent. CYP94B3 and CYP94C1 overexpression enhances a metabolic route leading to increased 12-*O*-Glc-JA and 12-HSO_4_-JA formation that does not involve free 12-OH-JA accumulation. *IAR3* and *ILL6* expression is also induced in infected leaves, and these amidohydrolases may be recruited in OE plants to allow the formation of additional 12-*O*-Glc-JA and 12-HSO_4_-JA from 12-OH-JA-Ile. Such a route probably implies an uncharacterized UDP-glucose glucosyltransferase (UGT) and the sulfotransferase ST2a. JA-Ile signalling activity is strongly reduced by hydroxylation and abolished by further oxidation to 12-COOH-JA-Ile. In addition to *JAZ* induction as a negative-feedback loop, hormonal oxidation also reduces the timing and amplitude of the pathway defence output. (This figure is available in colour at *JXB* online.)

Most studies on hormone homeostasis and interactions in plant defence have focused on biosynthesis and signalling (reviewed by Hoffmann *et al.*, 2011; Robert-Seilaniantz *et al.*, 2011; Pieterse *et al.*, 2012; Gimenez-Ibanez and Solano, 2013), but very little is known on how catabolic pathways intervene to generate appropriate hormone dynamics and govern precise dosage and duration of defence responses. Here, we investigated how the defence hormone JA-Ile, which is critical in resisting attack by *B. cinerea*, is turned over and how its oxidative catabolism conditions the JA metabolic network and defence output. The use of synthetic standards allowed the quantification of seven jasmonates, including the recently characterized JA-Ile catabolites (Fig. 7). Kinetic studies revealed quantitative and qualitative differences with profiles recorded previously in the wound response (Heitz *et al.*, 2012), indicating that JA metabolic genes/enzymes and flux through specific biochemical steps undergo stimulus-specific regulation. Although unconjugated jasmonates were in similar nanomolar ranges in both responses, the three JA-Ile conjugates were much less abundant after infection than upon wounding, suggesting either lower synthesis or higher turnover in infected leaves. JA-Ile levels culminated in infected leaves at only about 1/15 of the level reached in wounded leaves, indicating that less JA may be conjugated towards JA-Ile after infection. A possibility is that the SA pathway, activated by *B. cinerea* but not by wounding, specifically represses the synthesis of JA-Ile conjugates. The rather severe wound stress procedure generally used results in a transient but vigorous JA-Ile pulse because of synchronous stimulation across the leaves. In contrast, growing fungal necrotic lesions inflict a continuous, asynchronous stimulation where a ring of cells at the infection front are in a given metabolic and signalling state, leading to a steady increase in metabolite abundance. The levels of 12-OH-JA-Ile were about one order of magnitude lower than those of 12-COOH-JA-Ile, meaning that 12-OH-JA-Ile is either further oxidized, consistent with predominant *CYP94C1* expression, or undergoes other modifications. Upon wounding, where *CYP94B3* expression dominates, both compounds accumulate at similar levels (Widemann *et al.*, 2013). 12-OH-JA (tuberonic acid) was the second most abundant jasmonate after JA in response to *B. cinerea*, and glucosylated and sulfated 12-OH-JA derivatives also accumulated. Overall, these data illustrate the dynamics of the complex JA profile triggered in response to *B. cinerea* infection. This JA signature is likely to be non-exhaustive; for example, some other JA–amino acid conjugates like JA–tyrosine or JA–phenylalanine also accumulate in *B. cinerea*-infected leaves (E. Widemann, unpublished data). These compounds are also potential CYP94 substrates, but they are inactive in promoting COI1–JAZ co-receptor assembly, and no signalling activity has yet been assigned to these compounds in *Arabidopsis*.

### CYP94B3 and CYP94C1 shape the jasmonate signature in response to *B. cinerea*

We have shown previously that the wound-induced accumulation of the oxidized compounds 12-OH-JA-Ile, 12-COOH-JA-Ile, 12-OH-JA, 12-*O*-Glc-JA, and 12-HSO_4_-JA is largely dependent on CYP94B3 and CYP94C1 activity on JA-Ile (Heitz *et al.*, 2012; Widemann *et al.*, 2013). Here, we established that *CYP94C1* has the strongest response to fungal attack, with the weakest responding gene being *CYP94B1*. CYP94B1 was recently characterized as a close relative to CYP94B3 with similar catalytic properties (Koo *et al.*, 2014). The metabolic impact and phenotypes were observed predominantly in plants strongly overexpressing CYP94B1, while a single *cyp94b1* mutant behaved like the WT. The work presented here dissected the contribution of the three JA-Ile oxidases to the JA signature by using a series of seven *CYP94*-modified plant genotypes. For most compounds, the triple *cyp94b1b3c1* mutant did not accentuate the profiles of the double *cyp94b3c1* mutant, consistent with a quantitatively minor role of CYP94B1 in this pathosystem. As an exception to this trend, an impact of *cyp94b1* mutation could be detected as a 40–50% loss of 12-*O*-Glc-JA in the triple *cyp94b1b3c1* mutant compared with the WT2 control, while levels were not affected in *cyp94b3c1* plants relative to the WT1 control. Therefore, the role of CYP94B1 is best visualized when it acts alone (in *cyp94b3c1* plants) and where it may specifically contribute to the biosynthesis of 12-*O*-Glc-JA.

Several important findings can be drawn on the regulation of the *Botrytis*-triggered jasmonate circuitry (Fig. 7): (i) Most obviously, the direct dependence of 12-OH-JA-Ile and 12-COOH-JA-Ile accumulation on CYP94B3 and CYP94C1 expression, respectively, was evidenced with antagonistic effects in mutant and OE lines, which ranged over three orders of magnitude in the case of 12-COOH-JA-Ile in CYP94C1-modified genotypes (Fig. 1). These data identify a major role for CYP94C1 in JA-Ile oxidation upon *B. cinerea* infection. (ii) Consistent with a metabolic block, JA-Ile levels were enhanced moderately by *cyp94* mutations relative to the WT, but, conversely, JA-Ile was only decreased (by half) in the B3-OE line. The C1-OE line used (C1-OE3B), although exhibiting reduced JA-Ile levels after wounding (Heitz *et al.*, 2012), featured essentially WT JA-Ile levels after *B. cinerea* in two independent experiments. C1-OE bore larger fungal lesions than WT, which may stimulate more tissue to accumulate JA-Ile levels comparable to WT in total leaves, but which may be reduced on a per cell basis and attenuate signalling. This interpretation would also hold true for B3-OE, but one should keep in mind that B3 enzyme acts primarily on JA-Ile, while C1 enzyme readily uses 12-OH-JA-Ile as substrate (Heitz *et al.*, 2012) and is less likely to directly affect JA-Ile levels. (iii) Examination of other JAs revealed that *B. cinerea*-induced 12-OH-JA and 12-*O*-Glc-JA accumulation is intact in *cyp94b3* or *cyp94c1* single or double mutants and therefore these compounds are unlikely to arise massively from the indirect oxidation-cleavage pathway via 12-OH-JA-Ile intermediate as after wounding (Widemann *et al.*, 2013). In this respect, the precise contribution of the amidohydrolase pathway upon infection needs to be examined in corresponding mutant lines. Another possibility would be that these compounds are formed from JA by successive oxidation and glucosylation by unknown enzymes (Fig. 7). In contrast, 12-HSO_4_-JA accumulation was clearly CYP94B3-dependent, pointing to its formation via 12-OH-JA-Ile through an unknown sequence of reactions. Unexpectedly, B3-OE and C1-OE plants had increased 12-*O*-Glc-JA and to a lesser extent 12-HSO_4_-JA levels, suggesting that CYP94B3 or CYP94C1 overexpression creates a new biosynthetic route leading to these compounds, and that this may not proceed through free 12-OH-JA accumulation. Clearly, mapping all possible metabolic conversion routes between jasmonates and their regulation under different stress or developmental conditions needs deeper investigation, including the characterization of new enzyme activities.

### Perturbed JA-Ile turnover suggests novel control points of antifungal defence and resistance

The general assumption is that biotic stress such as herbivory or infection by necrotrophic microbes triggers rapid JA-Ile hormone accumulation, and, in turn, a simple transcriptional model predicts that the defence response amplitude and subsequent resistance track hormone levels. This has been verified in many studies, but the present data identify new situations where more complexity emerges, and distorts the linear relationship between JA-Ile levels and defence/resistance strength.

Two distinct scenarios may underlie the characteristics of CYP94 KO and CYP94 OE plants: *cyp94* KO plants accumulate more JA-Ile due to reduced catabolism, yet they do not exhibit a clear gain in fungal resistance. Possibly, JA-Ile levels are close to saturation in terms of defence signalling, even in the WT, and a moderate hormone increase in KOs only generates a negligible increase in *PDF1.2* and *PR4* expression, insufficient to improve resistance. The transiently increased *ERF1* signal in KOs (2 dpi) confirmed a higher JA-Ile input, which was not translated into more defence. The inability of CYP94-deficient plants to durably deploy higher defence and resistance phenotypes may find an explanation in the prolonged overinduction of specific *JAZ* genes such as *JAZ1* and *JAZ5*. Although this has to be established at the protein and target promoter levels, it is likely that some *JAZ* genes react to abnormal JA-Ile increases in a negative-feedback loop to prevent excessive downstream target responses. As a consequence, increasing crop tolerance to necrotrophs by inhibiting JA-Ile catabolism will require more refined strategies.

In marked contrast, both OE plant lines showed clearly impaired antifungal resistance. In the case of B3-OE, this phenotype could be explained by reduced JA-Ile levels due to exacerbated catabolic inactivation that attenuates defence. The situation with C1-OE was more unexpected, as this line showed essentially normal JA-Ile accumulation, and depressed *JAZ8* and *JAZ9* expression, yet it bore the most severe *B. cinerea* symptoms and multiplication, along with largely impaired defence responses. The observation that specific *JAZ* genes are antagonistically affected when either blocking or enhancing *CYP94* expression suggests differential *JAZ* sensitivity to alterations in the hormone oxidation status. This may reveal a repression rheostat to allow flexible control in the signalling output by adapting JAZ repressor strength to actual JA-Ile turnover. Because OE plants are characterized by their high content of oxidized hormone, a possibility is that JA-Ile levels are not the sole hormonal factor determining defence output, and that the simultaneous presence of abundant 12-OH-JA-Ile and/or 12-COOH-JA-Ile catabolites negatively affects signalling through an unknown mechanism.

Another aspect revealed here concerns the interaction of JA-Ile catabolism with SA signalling. In OE plants, the JA signalling pathway is largely disabled, and consequently, this relieves the repression on the SA pathway, as attested by higher SA content and *PR1* marker expression. Predictively, this shift towards more SA defence in OE plants should lead to enhanced resistance towards biotrophic pathogens. This prediction is supported by the work of Hwang and Hwang (2010), who showed that *cyp94b3* mutants are more susceptible to the hemi-biotrophic bacterial pathogen *P. syringae* DC3000. In the same study, *Arabidopsis* plants ectopically expressing a pepper *CYP94B3* homologue were more resistant to the obligate biotrophic oomycete *Hyaloperonospora arabidopsidis* (Hwang and Hwang, 2010). These results show that perturbing JA-Ile turnover has distinct consequences on pathogens with different lifestyles.

### CYP94-mediated JA-Ile oxidation gradually impairs co-receptor assembly and signalling properties

Finally, we addressed the direct signalling capacities of JA-Ile and its two CYP94-generated derivatives by external treatment of seedlings, an approach that had not been explored so far. Hydroxylation was sufficient to remove most but not all gene-inducing activity, while the carboxy derivative lost any detectable activity. We cannot rule out that oxidation status could influence the uptake of compounds by seedlings. The most likely hypothesis is that oxidation alters the capacity of conjugates to act as ligands promoting co-receptor assembly and subsequent signalling, and this was supported by the *in vitro* pull-down assay with JAZ9, extending similar conclusions reported for other JAZ proteins (Koo *et al.*, 2014). The highly polar environment at the C12 carbon in 12-COOH-JA-Ile may prevent interaction with the hydrophobic pocket of the receptor that accommodates JA-Ile (Sheard *et al.*, 2010), but this needs experimental validation. We finally considered the possibility that an excess of oxidized derivatives may inhibit the signalling action of JA-Ile, providing a basis for understanding the impaired defence output in *Botrytis*-infected B3-OE and C1-OE plants, which overaccumulate these compounds. In preliminary experiments, concomitant application of JA-Ile along with an excess of oxidized derivatives did not strongly reduce JA-Ile gene-inducing activity. Therefore, understanding the molecular basis underlying impairment of JA-Ile-dependent defences in CYP94-OE plants will require further investigation.

## Supplementary data

Supplementary data are available at *JXB* online.

Supplementary Fig. S1. *IAR3* and *ILL6* amidohydrolase expression profiles in WT plants in response to *B. cinerea*.

Supplementary Fig. S2. Expression characteristics of B3-OE and *b1b3c1* mutant lines.

Supplementary Fig. S3. Expression profiles of the 12 *Arabidopsis JAZ* genes in WT plants in response to *B. cinerea* infection.

Supplementary Table S1. Primers used in this study.

Supplementary Data

## Abbreviations:

ANOVA: analysis of variance
dpi: days post-inoculation
ET: ethylene
FW: fresh weight
JA: jasmonic acid
JA-Ile: jasmonoyl-isoleucine
KO: knoc kout
OE: overexpressing
SA: salicylic acid
UPLC-MS/MS: ultra-performance liquid chromatography coupled to tandem mass spectrometry
WT: wild type.
