# Supplementary material for: CYP94-mediated jasmonoyl-isoleucine hormone oxidation shapes jasmonate profiles and attenuates defence responses to Botrytis cinerea infection
Source: J Exp Bot. 2015 Apr 22;66(13):3879–92. doi: 10.1093/jxb/erv190 (PMC4473988; doi:10.1093/jxb/erv190)
Supplement: Supplementary Data [file supp_66_13_3879__index.html]

CYP94-mediated jasmonoyl-isoleucine hormone oxidation shapes jasmonate profiles and attenuates defence responses to Botrytis cinerea infection — CYP94-mediated jasmonoyl-isoleucine hormone oxidation shapes jasmonate profiles and attenuates defence responses to Botrytis cinerea infection — Supplementary Data 

# CYP94-mediated jasmonoyl-isoleucine hormone oxidation shapes jasmonate profiles and attenuates defence responses to *Botrytis cinerea* infection

## Supplementary Data

Data files

**Files in this Data Supplement:**

- Supplementary Data - Supplementary Data
