# Supplementary material for: CYP94-mediated jasmonoyl-isoleucine hormone oxidation shapes jasmonate profiles and attenuates defence responses to Botrytis cinerea infection
Source: J Exp Bot. 2015 Apr 22;66(13):3879–92. doi: 10.1093/jxb/erv190 (PMC4473988; doi:10.1093/jxb/erv190)
Supplement: Supplementary Data [file supp_erv190_Fig_S1_S3_erv190.pdf]

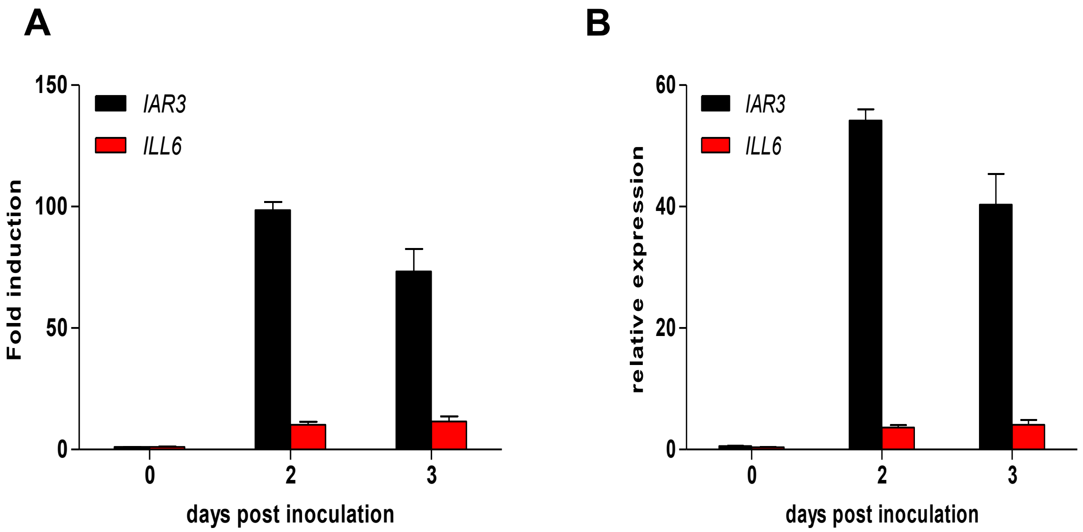

Figure S1. *IAR3* and *ILL6* amidohydrolase expression profiles in WT plants in response to *B. cinerea*. Wild-type (Col-0) leaves were harvested prior infection (0 dpi) and two or three days after inoculation (2 and 3 dpi, respectively) and submitted to RNA extraction. One µg of total RNA was reverse transcribed and expression of *IAR3 and ILL6* genes was determined by real-time PCR using gene specific primers and normalized with *EXP* and *TIP41* as reference genes. Histograms represent the mean ± SEM of three technical replicates. (A) Expression is represented as fold induction relative to expression level that was set to 1 for each gene at 0 dpi. (B) Relative expression of the amidohydrolase-encoding genes *IAR3* and *ILL6*, represented as gene-specific signal corrected by reference gene signal.

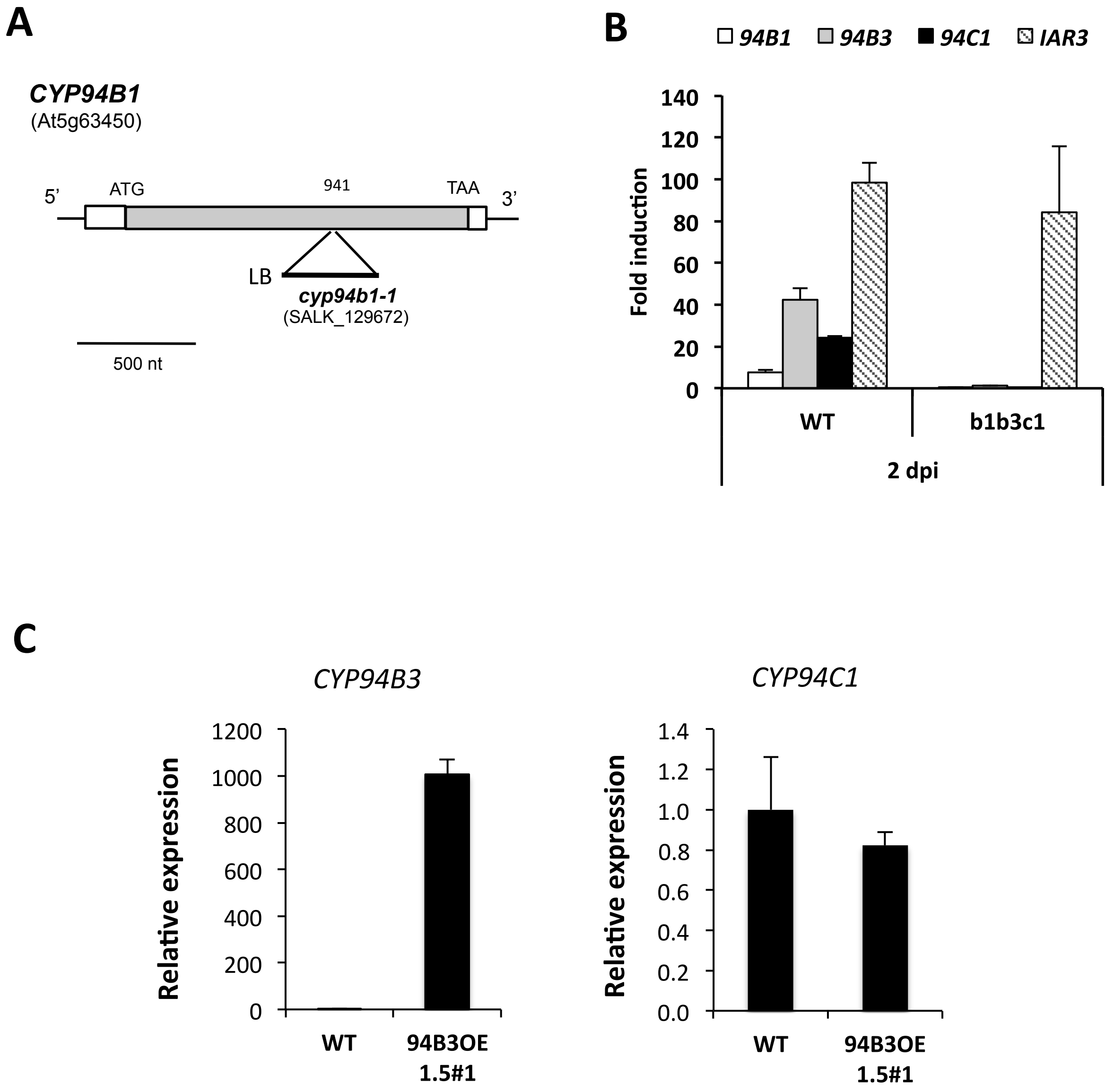

Figure S2. Characterization of *the cyp94b1b3c1* triple mutant and *CYP94B3*-overexpressing lines introduced in this study. (A) Schematic representation of the *cyp94b1-1* T-DNA insertion allele. White box represents the single exon with coding sequence in grey. LB : left border. (B) RT-qPCR analysis of gene 2 days post *Botrytis* inoculation (dpi), showing the absence of *CYP94B1*, *CYP94B3* and *CYP94C1* expression in triple *b1b3c1* mutant. *IAR3* expression was used as a stimulation control. (C) Basal expression of *CYP94B3* and CYP94C1 in wild-type (WT) and transgenic plants expressing a p35S::CYP94B3 construct (94B3OE 1.5#1). Expression in transgenic plants is relative to expression in WT that was set to 1. All data are means and SD from 3 determinations.

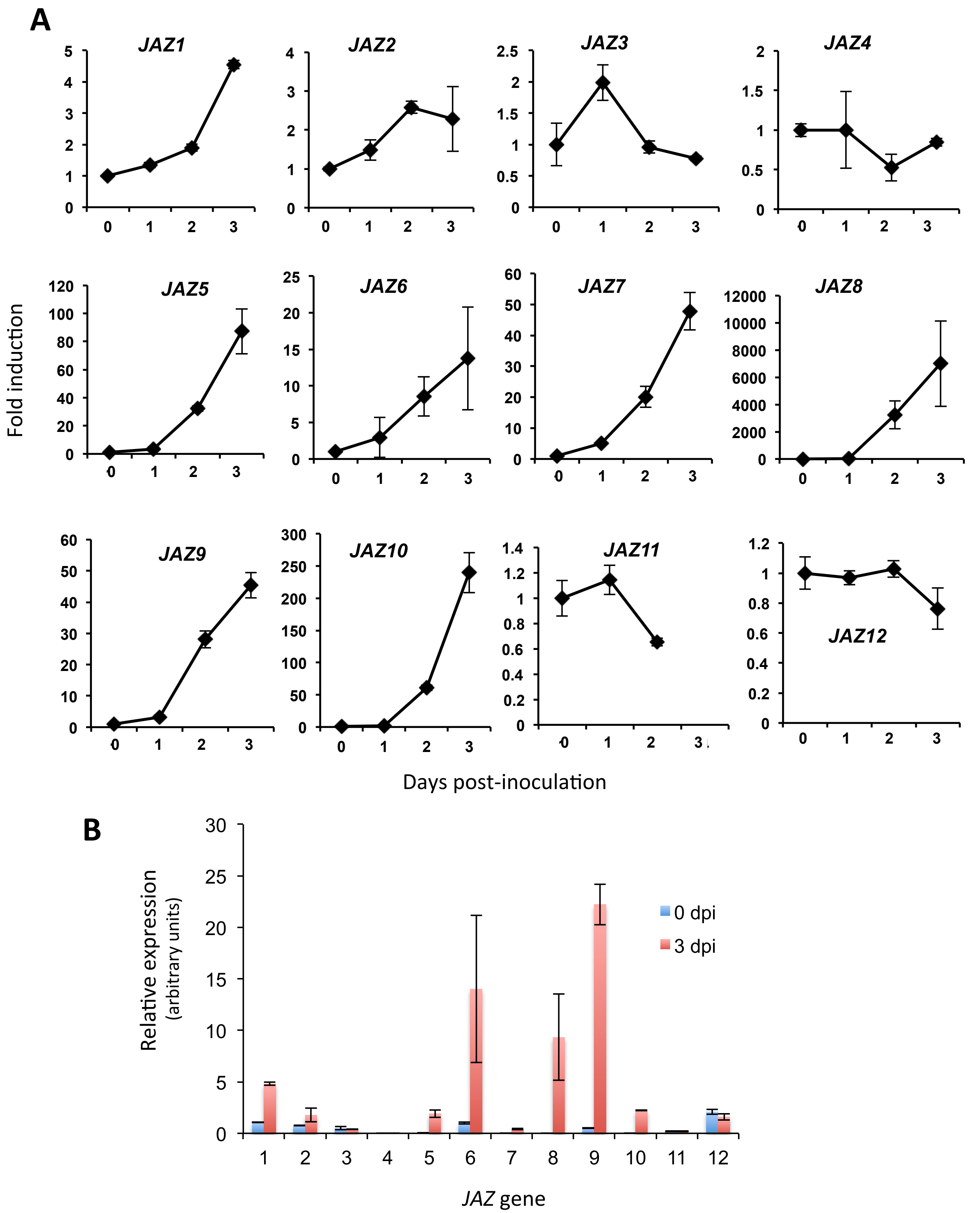

Figure S3. Expression profiles of the 12 Arabidopsis *JAZ* genes in WT plants in response to *B. cinerea* infection. Wild-type (Col-0) leaves were harvested prior infection (0 dpi) and 1, 2 or 3 days after inoculation and submitted to RNA extraction. One µg of total RNA was reverse transcribed and expression of *JAZ* genes was determined by real-time PCR using gene specific primers and normalized with *EXP* and *TIP41* as reference genes. Histograms represent the mean ± SEM of three technical replicates. (A) Kinetic expression represented as fold induction relative to expression level at T0 that was set to 1 for each gene. (B) Relative expression of *JAZ* genes at 0 and 3 dpi, represented as gene-specific signal normalized by reference gene signal.
